# A Constraint Condition for Foraging Strategy in Subterranean Termites

**DOI:** 10.1673/031.010.14106

**Published:** 2010-09-10

**Authors:** Wonju Jeon, Sheon-Young Kang, Nan-Yao Su, Sang-Hee Lee

**Affiliations:** ^1^ Division of Fusion and Convergence of Mathematical Sciences, National Institute for Mathematical Sciences, Daejeon, South Korea; ^2^ Department of Entomology and Nematology, Ft. Lauderdale Research and Education Center, University of Florida, Ft. Lauderdale, Florida, USA

**Keywords:** Subterranean termite, foraging efficiency, network connectivity, foraging strategy

## Abstract

Previous studies have explored the relationship between termite branch tunnel geometry and foraging efficiency in a model simulation in which foraging efficiency, *γ,* for two termite species, *Coptotermes formosanus* Shiraki and *Reticulitermes flavipes* (Kollar) (Isoptera: Rhinotermitidae), was investigated in response to two variables, the probability of tunnel branching (*P_branch_*) and the probability of tunnel branch termination (*P*term). It was found that simulated tunnel patterns based on empirical data did not have maximum foraging efficiency. We hypothesized that termites could increase their foraging efficiency in response to landscape heterogeneity. The present study investigated how termites could control the two variables, *P_branch_* and *P_term_*, in response to the external environment in terms of tunnel network connectivity. It was found that the best simulated strategy for *C. formosanus* and *R. flavipes* termites would occur if both *P_branch_* and *P_term_* were increased together. This study provides possible mechanisms for foraging strategies in subterranean termites and a baseline for future empirical work.

## Introduction

Animals must forage efficiently for food resources, or they risk a loss of fitness because energy that could be allocated to reproduction is used for survival (Werner and Mittelbach, 1981; Zimmer-Faust, 1987). A wide range of strategies could be used to achieve foraging efficiency by foragers, depending on the environment. Foraging strategies are important for understanding species survival in terms of foodweb structure and ecosystem function and the relationship between species biodiversity and ecosystem stability (Launchbaugh 1996). Foraging strategies have been studied both theoretically (Okubo 1980; Shlesinger et al. 1995; Diekmann et al. 2000) and experimentally (Cain et al. 1985; Forcardi et al. 1996; Gillingham and Bunnell 1989).

Previous studies have contributed to an understanding of foraging behavior (i.e., searching for and consuming food) from a cost-benefit perspective. However, these studies have only investigated foraging in open terrestrial environments. The foraging strategies of subterranean foragers, such as termites, differ in that they are highly constrained by the structural design of the substrate in which the foragers move (Lee et al., 2008a). These constraints cause an increase in the number of variables that could impact foraging. The foraging behavior of subterranean termites has been studied using monitoring stations combined with markrelease-recapture methods (Grace et al. 1989; La Fage et al. 1973; Su et al. 1984). However, the information derived from these investigations has been insufficient to provide an understanding of subterranean termite foraging strategy (Gallagher and Jones 2005). Lee et al. (2006, 2007a) studied the foraging strategy of subterranean termites and constructed a lattice model to simulate termite tunnel networks for *Coptotermes formosanus* Shiraki and *Reticulitermes flavipes* (Kollar). The model was based on experimental data obtained from homogeneous soil substrates without food resources (Su et al. 2004). The same model was used to examine the relationship between foraging efficiency and tunnel network geometry as a way of understanding how termites could maximize foraging efficiency (Lee et al. 2006, 2007a, 2007b, 2009a).

Following these studies, Lee et al. (2009b) conducted model simulations using experimental values for the probability of tunnel branching, *P_branch_*, and the probability of branch tunnel termination, *P_term_*. Foraging by termites in tunnels that were based on experimental values was less efficient than in tunnels based on other values of *P_branch_* and *P_term_*. These results suggested that termites may regulate *P_branch_* and *P_term_* in order to increase the foraging efficiency in response to landscape heterogeneity.

Lee et al. (2008b, c) found evidence for the hypothesized role of landscape heterogeneity by showing experimentally that termites began tunnel excavations only at sites with surface irregularities, which are a major factor in landscape heterogeneity.

Because termites have limited energy available for digging tunnels and foraging, if termites construct too many branching tunnels in regions with high heterogeneity, the energy available for foraging could be reduced, which is likely to lead to a decrease in foraging efficiency. Thus an additional constraint condition seems to be needed to restrict branching and the occurrence of long branched tunnels. This constraint condition can be described by the two variables, P_branch_ and P_term_.

The purpose of the present study is to explore further the effects of these two variables, *P_branch_* and *P_term_*, on foraging efficiency because these variables can reveal essential features of the termite tunnel network (Hedlung and Hederson 1999). Specifically, the goal is to understand how *P_branch_* and *P_term_* can be optimized for higher foraging efficiency in relation to tunnel network connectivity.

## Methods

The connectivity of a termite tunnel network was used as the constraint condition for values of *P_branch_* and *P_term_*. The network connectivity was characterized by the algebraic connectivity, σ, defined as the second-smallest eigenvalue of the Laplacian matrix of the network (Fiedler 1973; Grone and Merris 1987). Given a network with node set *V* = {*v_1_*,*V_2_*,…, *V_n_*} and a link set between the nodes, its Laplacian matrix *L* is defined as (Merris 1998):

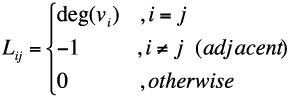

where deg(*v_i_*) denotes the degree of the *i*-th node, which is defined as the number of links emerging from the *i*-th node. The terms *i* and *j* represent the *i*-th and *j*-th nodes, respectively (*i,j* = 1,2,3,…,*n*).

**Figure 1.**
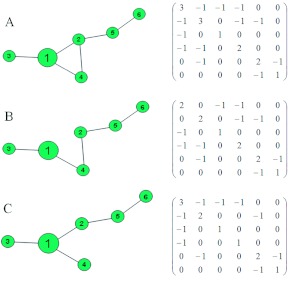
Example networks with simple connections and their Laplacian matrices. High quality figures are available online.

The algebraic connectivity, σ, reflects how well the overall network is connected. In order to facilitate an understanding of connectivity, three simple networks and their corresponding Laplacian matrices are given in Figure 1 as examples. The eigenvalues for Figure 1 A are listed as {0.0000, 0.4131, 1.1369, 2.3595, 3.6977, 4.3928}, while the eigenvalues for graphs B and C are {0.0000, 0.2679, 1.0000, 2.0000, 3.0000, 3.7321} and {0.0000, 0.3249, 1.0000, 1.4608, 3.0000, 4.2143}, respectively. The value of σ is highest for network A, which is apparent from a visual comparison of the three examples.

**Figure 2.**
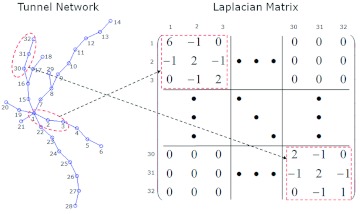
A typical termite tunnel pattern of *Coptotermes formosanus,* consisting of 32 nodes and 31 links and the network's Laplacian matrix. High quality figures are available online.

Figure 2 is a schematic representation of a tunnel network of *C. formosanus*. The tunnel network was simulated using the lattice model proposed in Lee et al. (2009b). The schematic consists of 32 nodes, 31 links, and the network's Laplacian matrix.

In the tunnel network, the nodes represent the end points of the tunnel segments, defined as the line connecting the two closest points of a tunnel that did not deviate from the tunnel path (Su et al. 2004). In the simulation model, when a developing tunnel crosses with other tunnels, nodes are not created because different tunnels tend to occur at different depths below the ground surface. Thus, although termite tunnel networks with many branching tunnels may appear to have complex connectivity among tunnels, their actual connectivity can be low.

## Results

**Figure 3.**
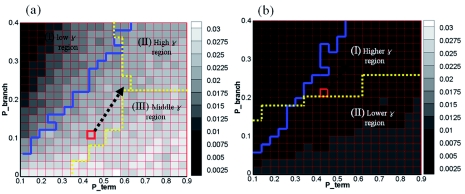
The map of algebraic connectivity, σ, for the branching probability, *P_branch_*, and the branching termination probability, *P_term_*, for (a) *Coptotermes formosanus* and (b) *Reticulitermes flavipes.* Darker shades of gray correspond to lower values of connectivity. The solid blue line separates domains of lower and higher σ values, and the yellow dotted line divides the map into regions with low, middle, and high foraging efficiency, *γ*, respectively. The red box indicates the value of *γ* for empirical tunnel patterns. High quality figures are available online.

Connectivity for 1,000 simulated tunnel networks of *C. formosanus* and *R. flavipes* was calculated for different values of *P_branch_* and *P_term_* (Figure 3).

For *C. formosanus* (Figure 3 a), σ was higher with a higher *P_term_* and a lower *P_branch_*, while σ decreased with an increase in *P_branch_* and a decrease in *P_term_*. Lee et al. (2009b) showed that there are three distinct regions in a map of foraging efficiency, *γ*, according to *P_branch_* and *P_term_*. The distinct regions are indicated in Figure 3 by the yellow dotted line. In contrast, the connectivity map was separated into two domains, an upper and lower, as indicated by the solid blue line. Silhouette analysis was used as the separation method for the σ map (Rousseeuw 1987). This analysis showed that when the σ map was partitioned into two regions, the optimization score was highest in the case of *C. formosanus* (Table 1). The *k*means algorithm (Hartigan and Wong 1979) was then used to divide the σ map with *k* = 2. These divisions are indicated by the blue solid lines in Figure 3 (a). The values of σ were markedly lower in the upper domain than in the lower domain.

Lee et al. (2009b) hypothesized that termites control the values of *P_branch_* and *P_term_* in response to landscape heterogeneity in order to move the red box from region I to region II (Figure 3 a). The connectivity map gives a constraint condition for moving the red box. The best strategy for termites would be to increase *P_branch_* and *P_term_* together in the direction of the arrow within the lower connectivity domain.

These data suggest that very low connectivity leads to an increase in traveling cost during foraging due to numerous tunnel intersections that termites have to select depending on their direction of movement. On the other hand, very high connectivity results in a decrease in *γ*, because the short and infrequently branching tunnels result in the termites to covering an area that it is insufficient to meet their foraging needs (see Figures 1 and 2 in Lee and Su 2009b).

**Table 1.**
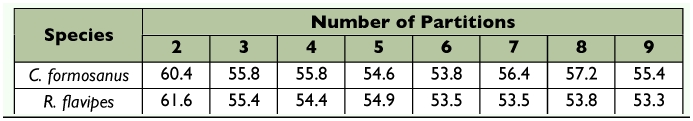
Silhouette score values for each partition

Like the σ map of *C. formosanus,* the σ map for *R. flavipes* was divided into two domains, an upper and lower (Figure 3 b). The red box was located at *P_branch_* = 0.21 and *P_term_* = 0.47. According to Lee and Su (2009b), increasing *P_branch_* and decreasing P_term_ could increase foraging efficiency. However, in terms of network connectivity, increasing both *P_branch_* and *P_term_* within the lower σ domain in order could avoid lower connectivity.

## Discussion

Lee et al. (2007a, 2009b), showed that simulated termite tunnel patterns, based on experimental data obtained from homogenous sand substrates, maximize their foraging efficiency defined as the ratio of energy gain for obtained food to loss for transporting food for a given time. In the present study the goal was to examine how termites construct their tunnel networks in heterogeneous landscape and determine if this would optimize their survival probability in a cost-benefit perspective. From the fact that termites begin their tunneling behavior only at surface irregularity, high heterogeneity is likely to cause many tunnel branching because the degree of surface irregularity increase with the heterogeneity (Lee et al., 2008c). When tunnel branching occurs, tunnel intersections are generated at the branching site. Termites frequently encounter tunnel intersections while foraging or moving within their networks. The direction chosen by termites at tunnel intersections is likely to affect foraging efficiency because the path length between food resources and the nest can vary significantly. Thus, the number of tunnel intersections is related to tunnel network connectivity, and connectivity is likely to be an important factor in foraging efficiency.

The network patterns were characterized by two variables, the probability of tunnel branching, *P_branch_*, and the probability of tunnel branch termination, *P_term_*, because these variables capture essential features of the termite tunnel network (Hedlung and Hederson 1999). The simulation showed that the two termite species could increase *P_branch_* and *P_term_* together to achieve intermediate connectivity and thereby improve foraging efficiency.

The results may be inconsistent with field observations because there may be other constraint conditions such as soil hydrology or soil particle size associated with the physical environment. Soil characteristics may, in turn, interact with resource abundance to affect tunnel search patterns. For instance, termites could decrease tunnel growth in areas with poor soil conditions and increase the extent of tunnels in other areas. Although the simulation may not provide exact predictions of termite behavior in the field, the results of this study provide insights into the foraging strategy that could be used by termites to improve foraging efficiency. The results also suggest directions for future empirical investigations of termite foraging strategy.
